# A comprehensive mapping review of participatory autism research over a 22-year period

**DOI:** 10.1186/s40900-026-00998-y

**Published:** 2026-11-02

**Authors:** Diana Weiting Tan, Jacquiline den Houting, Marion Rabuka, Hannah Winspear-Schillings, Tori Haar, Elizabeth Pellicano

**Affiliations:** 1 Macquarie School of Education, Macquarie University, Sydney, Australia; 2 School of Psychological Science, University of Western Australia, Perth, Australia; 3 Olga Tennison Autism Research Centre (OTARC), La Trobe University, Melbourne, Australia; 4 Department of Clinical, Education and Health Psychology, University College London, London, UK

**Keywords:** Autism, Participatory research, Meta-science, Mapping review, Community involvement

## Abstract

**Background:**

Participatory research, which involves community members as partners rather than merely research subjects, has gained prominence in autism research. However, the extent and nature of participatory approaches across the field remain unclear.

**Methods:**

We conducted a mapping review of autism research reporting community involvement, searching six databases. We coded who was involved, at what level, and in which research activities, and examined factors associated with participation.

**Results:**

We identified 382 studies published between 2001 and 2023, with 95% published in the past decade. Two-thirds (67%) involved Autistic people, though one-third claiming to be “participatory” excluded them entirely. The most common participation level was consulting (31%), with community-led (5%) and community-controlled (1%) research remaining rare. Community involvement concentrated in study design (73%) rather than analysis (33%) or dissemination (29%). Participatory research was heavily concentrated in Western countries (USA, UK, Australia, Canada: 86%), applied research areas, and qualitative studies. Deeper participation was associated with identity-first language use and publication in journals with clear community-centered agendas.

**Conclusions:**

While participatory language is increasingly becoming the norm in autism research, genuine power-sharing between researchers and community members is still a rare occurrence. Most participatory research operates at consultative levels, with academic researchers retaining primary decision-making authority. Moving toward authentic participation requires systemic changes to funding structures, institutional policies, and epistemic hierarchies that determine whose knowledge counts.

**Supplementary Information:**

The online version contains supplementary material available at https://doi.org/10.1186/s40900-026-00998-y.

## Plain language summary

In this review, we looked at 382 autism studies published between 2001 and 2023 that claimed to involve the community (Autistic people, family members, etc.) as collaborators rather than participants providing data for the studies. We checked who was involved, how much say they had, and what parts of the research they helped with.

What we found:

- Almost all this research (95%) has happened in just the last decade showing that participatory approaches are a recent trend.
- One in three studies calling themselves “participatory” didn’t actually include Autistic people at all.
- Most “involvement” was quite shallow. Researchers mainly *consulted* the community, rather than sharing real decision-making power. Studies that were genuinely community-led (5%) or community-controlled (1%) were rare.
- Community members were mostly brought in to help design the study (73%), but far less often involved in analysing results (33%) or sharing findings (29%) where influence is important.
- This kind of research is often found in wealthy Western countries (US, UK, Australia, Canada made up 86%), and tends to show up more in applied, qualitative studies than in other types.
- Deeper, more genuine involvement was more likely in studies that used identity-first language (e.g., “Autistic person”) and in journals with an explicit community-focused mission.

Talking about “participatory research” has become common in autism research, but power sharing with the Autistic and autism communities is still rare. Researchers usually stay in control. To change this, we need bigger shifts in recognising whose knowledge and expertise gets valued and taken seriously.

**Supplementary Information:**

The online version contains supplementary material available at https://doi.org/10.1186/s40900-026-00998-y.

## Background

Autism research has historically excluded the very people it seeks to understand [1–3]. This history has prompted sustained critique from Autistic scholars, advocates, and community members, who have questioned not only the relevance and impact of autism research, but also its ethical legitimacy and epistemic foundations [4–8]. Consequently, many Autistic people have experienced autism research as “out-of-touch with the ‘real’ world” [9], misaligned with community priorities and everyday life [6, 9, 10]. In response to these critiques, *participatory research* has gained increasing prominence within autism research as a way of reconfiguring – and, in some cases, repairing – relationships between academic researchers and the communities their work concerns [11–15]. In this article, we use participatory research as an umbrella term for “a school of approaches that share a core philosophy of inclusivity and recognizing the value of engaging in the research (rather than including only as subjects of the research) those who are intended to be the beneficiaries, users, and stakeholders of the research” [16].

Historically, participatory research draws on two overlapping traditions. The *Northern tradition*, rooted in Kurt Lewin’s action research in the 1940s in the USA and UK, conceptualised research as an iterative cycle of planning, action, and reflection aimed at addressing practical problems [17]. Methodological approaches emerging from this tradition tend to emphasise collaborative problem-solving and the generation of knowledge that is directly usable for policy and practice [18]. In contrast, the *Southern tradition* emerged in the 1970s in Latin America, Africa, and Asia within contexts of colonisation and deep socio-economic inequality [18]. Strongly influenced by Paulo Freire’s pedagogy of liberation [19] and the work of activist scholars like Orlando Fals Borda [20], this tradition frames participatory research as a political pursuit – a means for marginalised groups to analyse their reality and act collectively toward emancipation. Approaches associated with this tradition place particular emphasis on the participation and leadership of marginalised communities across all stages of the research process [18]. Contemporary participatory research often integrates elements of both traditions, spanning a continuum from more practice-oriented, problem-solving approaches to more explicitly social-movement-oriented work [18]. For this review, we adopt an inclusive stance towards what constitutes participatory research, encompassing both traditions across this continuum.

Variations in participatory research approaches are characterised across three dimensions of how participation is enacted in the current review. The first dimension concerns *who* is involved. Cargo and Mercer [16] describe five types of community members: (a) the ultimate beneficiaries of the research (in the case of autism research, Autistic people); (b) the interpersonal support network of those beneficiaries (i.e., family members and friends of Autistic people); (c) those who interface directly with the previous two groups (e.g., clinicians, educators, and other service providers), (d) those who operate at broader systems levels, including policy makers and executive directors, and (e) interested members of the general public. It is important to note that these groups are not interchangeable. Autistic people are the ultimate beneficiaries of autism research and hold lived experience that no other non-autistic groups share, whereas other non-autistic stakeholder groups contribute proximal, professional, or institutional perspectives that may complement – but cannot replace – Autistic people’s own experiences. As in Tan et al. [21], we use “community involvement” to refer to the involvement of any of the community members described by Cargo and Mercer but distinguish the involvement of Autistic people from that of non-autistic stakeholders throughout this review.

The second dimension is *depth*, which refers to the degree to which decision-making power is shared with community members. Drawing on Arnstein’s [22] ladder of citizen participation, and its subsequent adaptation for autism research [23], levels of participation are commonly described as informing, consulting, engaging, co-producing, community-led, and community-controlled ([21]; see Table 1). Based on this framework, informing, consulting, and engaging are considered tokenistic forms of involvement, wherein academic researchers retain primary decision-making authority; by contrast, co-producing, community-led and community-controlled approaches reflect genuine power-sharing, wherein community members hold equal, substantive, or full authority over research decisions. The third dimension is *breadth*, referring to the range of research activities and stages in which community members are involved, such as agenda setting, study design, data collection, analysis, and dissemination [23, 24].

While flexibility across these dimensions allows participatory approaches to be adapted to different research contexts, it also means that researchers may differ substantially in how they understand and operationalise participation [23, 25], resulting in considerable variation in what is construed as participatory research [23, 26]. In some cases, consultative forms of involvement are described using the same terminology as approaches involving shared decision-making [27, 28]. This lack of conceptual clarity risks conflating different forms of engagement and obscuring tokenistic practices under a participatory label. Importantly, community members have described experiences of “consultation fatigue” arising from repeated involvement in processes that offer limited influence or impact [28–30]. Taken together, these concerns point to an ethical and empirical imperative to examine participatory autism research practices. As yet, however, no systematic analysis has examined how these approaches are actually enacted across the field, leaving it unclear whether participatory rhetoric has translated into meaningful shifts in research practice.

**Table 1:** Characteristics of each of the eight rungs of the participatory ladder based on Arnstein [22], reproduced with permission from Tan et al. [21]

Level of participation	Characteristics
Community controlled	Community members have full decision-making and negotiation power over all aspects of the research. Participation at this level signifies the highest level of power redistribution, with community members holding all the decision-making power.
Community led	Community members have dominant decision-making authority over all aspects of the research. Participation at this level signifies a high level of power redistribution, with community members holding more decision-making power than researchers.
Co-producing	Community members and researchers share planning and decision-making responsibilities across all aspects of the research. Participation at this level signifies some level of power redistribution, with community members holding equal decision-making power as researchers.
Engaging	Community members are invited to advise on research, but researchers retain the right to determine the legitimacy or feasibility of the advice. The breadth of participation is also limited to only a few aspects of the research (e.g., reviewing procedure and participant-facing materials). Participation at this level is tokenistic with researchers retaining more decision-making power than community members.
Consulting	Community members are invited to voice their opinions, but it is not clear whether researchers have taken them into account. The breadth of participation is also limited (e.g., only reviewing participant-facing materials). Participation at this level is tokenistic with researchers retaining most decision-making power.
Informing	Community members are informed about decisions made in research, but there is no channel for feedback and no power for negotiation. Participation at this level is tokenistic with researchers retaining full decision-making power.
Educating	Community members are educated about research to “correct” their “misconceptions” about research, rather than being genuinely involved. There is no participation at this level.
Coercing	Community members are misled and manipulated into being involved in research for the purpose of optics. There is no participation at this level.

Note. In this review, we defined community members as individuals from the Autistic community, including lay Autistic people and Autistic researchers, and the broader autism community, including family members of Autistic people and professionals. This approach reflects current understanding in the field and acknowledges the different perspectives that various community members bring. There is, however, a need to shift the power in shaping research processes specifically to Autistic community members, particularly lay Autistic people outside of formal academic institutions (see ‘Discussion’)

### Factors shaping participatory autism research

The growing prominence of participatory approaches in autism research can be attributed to several factors. First, sustained advocacy from Autistic community members has been foundational in calling for greater participation in research [5, 8, 31]. Second, the increasing visibility and leadership of Autistic researchers within academia has contributed to shifts in how research is conceptualised, conducted, and evaluated [32–35]. Autistic autism researchers bring lived experience expertise into academic discourse and challenge conventional researcher-participant boundaries. In some accounts, they are described as potentially acting as intermediaries between academic research and non-academic Autistic community members, helping to create conditions that may support more inclusive forms of participation [1, 21].

Third, academic journals have played a significant role in promoting participatory research through editorial policies encouraging or mandating the reporting of community involvement [36, 37]. In 2015, *The British Medical Journal (BMJ)* required authors to include a Patient and Public Involvement (PPI) statement in all research articles. One year after implementation, *The BMJ* saw a 10-fold increase in articles reporting PPI, from 0.1% to 11% [38]. Following this precedent, *Autism*, one of the leading journals in autism research, introduced mandatory reporting of community involvement in 2021, though this requirement was later rescinded in 2024 [39]. Tan and colleagues [21] evaluated the impact of this policy and reported a five-fold increase in the reporting of community involvement, from 10% to 55%. The authors further characterised the nature and extent of participatory approaches and found that community members tended to be involved primarily in consultative capacities, with participation occurring more frequently in applied rather than basic research – a finding replicated in a recent review of participatory approaches in genetic research [40]. There was also an over-representation of participatory autism research in Western countries [21]. Nevertheless, Tan et al.’s findings were restricted to one autism-specific journal. Hence, the overall trends and characteristics of participatory research across the broader autism field remain unknown.

### Existing reviews

To date, several systematic or scoping reviews have examined aspects of participatory autism research. Jivraj and colleagues’ [41] scoping review examined the influence of participatory research on the research process and outcomes in neurodevelopmental research. The authors identified just seven articles, of which two focused on autism. This limited yield, however, was likely due to the strict inclusion criterion requiring detailed descriptions of how partnerships were established, implemented, and evaluated, rather than an indication of the rarity of participatory autism research. While this criterion was necessary to address the review’s specific question, it limited comprehensiveness. Other reviews have focused on specific methodologies, such as participatory approaches in interpretative phenomenological analysis [42], or specific domains of autism research, including genetics [40] and Autistic young people’s involvement in education research [43]. Though valuable, these reviews do not capture the full range of participatory claims across autism research, nor do they systematically examine variation in the degree, breadth, or timing of community involvement. As a result, the literature remains fragmented, making it difficult to draw field-level conclusions about how participatory approaches are being enacted.

### The current review

As participatory language becomes increasingly normative within autism research, there is a growing need to take stock of how participation is understood and implemented in practice. The current review examines the extent and nature of participatory approaches within autism research by systematically mapping participatory practices across the field. In doing so, we aim to contribute to greater conceptual clarity and to support more transparent, reflective, and effective participatory autism research. Specifically, this review addresses three main research questions:

1. Has community involvement in autism research increased over time?
2. What are the characteristics of participatory autism research, including who is involved, at what level of participation studies operate, and the breadth of community members’ involvement?
3. What factors may be associated with greater community involvement (e.g., research area, methods, funding source)?

## Method

We first searched the Cochrane Library and PROSPERO databases to confirm that no other systematic reviews of the current topic existed or were in progress before developing the current review.

### Transparency and openness

All data, analysis codes written in RStudio [44], research materials (including our coding scheme), an interactive dashboard, and preregistered review protocol are available at https://osf.io/aw4km/overview?view_only=84b23759419a4b36864a3f4c8b9f70b6. The review protocol was preregistered on PROSPERO (CRD42021293592). Deviations from this preregistration include: (a) the exclusion of the research question on the quality of reporting community involvement due to time constraints, and (b) two unplanned analyses on the impact of participatory approaches on language use and journals in which studies were published (rationale detailed in the relevant section below). This review was originally conceived as a systematic review. During peer review, however, it was noted that the review’s aim align more closely with a mapping review, as defined by Campbell et al. [45]. We agree with this assessment and have revised the manuscript to describe this work as a mapping review throughout, while retaining the systematic search and screening procedures originally deployed. To support broader engagement with the dataset, we developed an interactive dashboard allowing readers to explore the data further (https://dianawtan.github.io/sys-rev-par/). Claude [46] was used in developing the dashboard but not in the preparation of this research. We adhered to the Preferred Reporting Items for Systematic reviews and Meta-Analyses (PRISMA) guidelines for systematic reviews [47] which is consistent with current reporting recommendations for mapping reviews [48].

### Search strategy

We searched the following electronic databases: PsycINFO (Psychology), Scopus (Multidisciplinary Sciences including Social Sciences), PubMed (Life Sciences and Biomedical), MEDLINE (Multidisciplinary Medical Sciences), EMBASE (Biomedical and Pharmacological), and ERIC (Education). In collaboration with a university librarian and Autistic advisors, a structured search strategy was developed and adapted for each database (see Table S1).

Autism-related terms were searched in the title, abstract, and keyword fields and included: (autis* OR asperger* OR (pervasive AND development*)). As community involvement and participatory approaches are often described within manuscript bodies rather than indexed fields, participatory research search terms were searched across all available fields. These terms captured participatory approaches (e.g., *community-based participatory research; participatory*,* collaborative*,* action*,* co-produced*,* co-design*,* user-led*,* or inclusive research*), as well as community involvement (e.g., *lived experience*, *consumers*, *patients*, *participants*, *parents*, *families*, *caregivers*, *stakeholders*, or *advocates* in partnership with *researchers*). Proximity operators (e.g., within five words) and database-specific syntax were used where available to maximise sensitivity. No date restrictions were applied. The initial search was conducted in August 2021 and updated in September 2024 to capture studies published in 2022 and 2023.

### Inclusion and exclusion criteria

We included any peer-reviewed empirical studies that employed quantitative, qualitative or mixed-method designs. Single-case studies, reviews of any kind (e.g., systematic reviews), conference abstracts, dissertations, editorials, commentaries, letters, books, and articles not available in English were excluded. At the time of the review, the research team decided to exclude single-case studies as they are typically structured around the study of one participant, involving researcher-driven decisions specific to that individual [49], thus offering limited scope for collaborative decision-making with the community. We note an emerging proposal to combine participatory approach with single-case experimental designs [50], though, at present, this proposal remains largely conceptual.

### Article selection

**Fig. 1:**
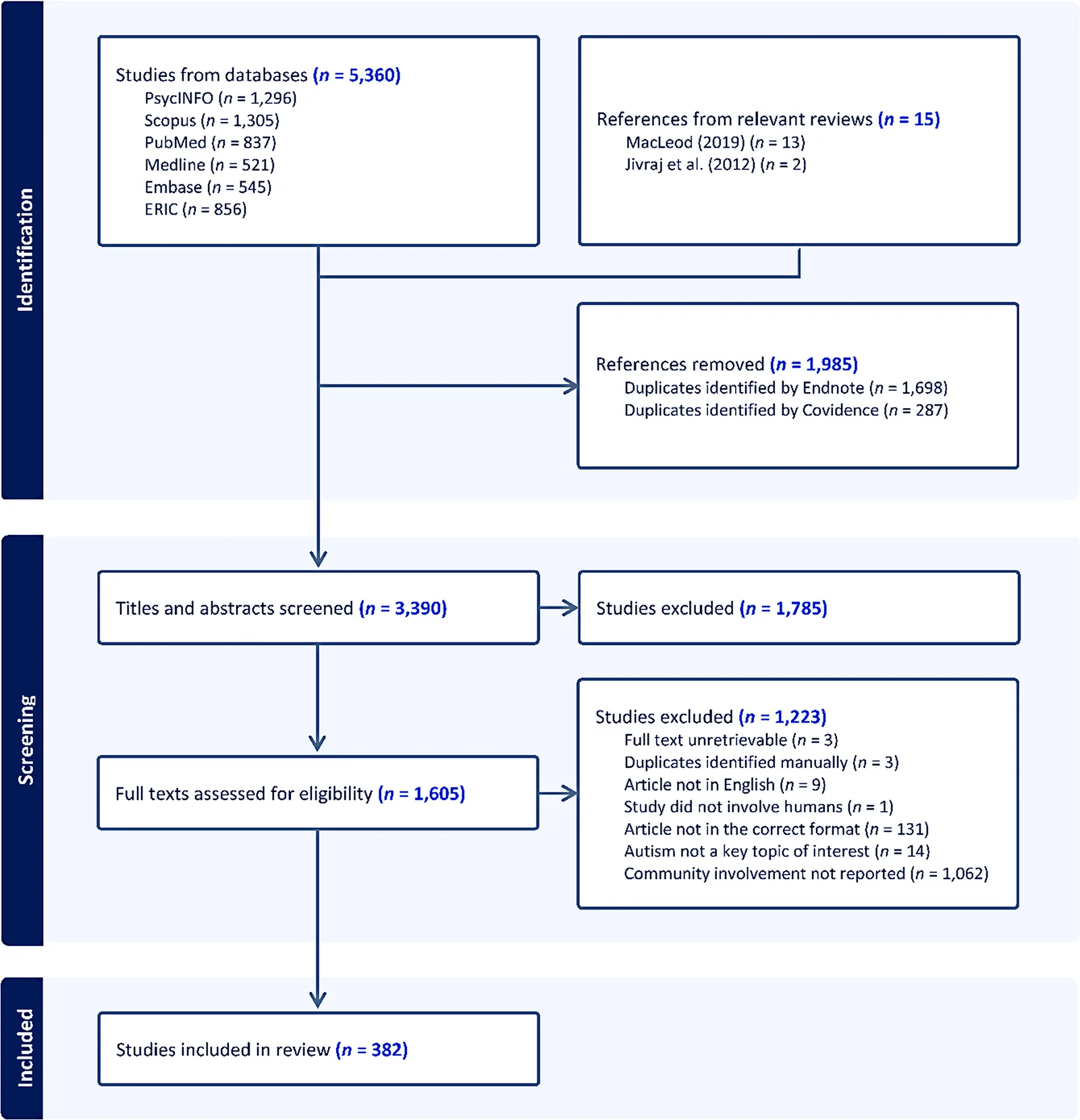
PRISMA flow chart

All search results were imported into Covidence [51] for screening. DT screened all the search results by title and abstract to include empirical studies related to autism. MR, HW-S, and three other volunteers independently double screened 30% (*n* = 1,017) of the titles and abstracts with substantial agreement (range of Cohen’s κ = 0.70–0.85) [52]. Next, the full texts of the potentially eligible articles were retrieved and reviewed by DT to include empirical studies that described a participatory approach or community involvement. 80% (*n* = 1,285) of the full texts were independently double screened by MR, HW-S, TH, and five other volunteers with substantial-to-almost-perfect agreement (range of Cohen’s κ = 0.67–0.99). All screening discrepancies were resolved through discussions. Figure 1 shows the details of the studies screened and included at each stage.

### Data extraction and coding

Every article was read in its entirety (including acknowledgements and contribution statements) and coded by DT using a bespoke data extraction template developed in Covidence. Half (50%) of the articles (*n* = 191) were double coded by five volunteers and verified by DT. General information about the included articles were extracted, including: (i) journal name; (ii) language use (e.g., identity-first or person-first language or combination of both); (iii) country where the study was conducted, based on the corresponding author’s location; (iv) research topic^1^ (e.g., biology, services) based on the descriptions outlined in the Interagency Autism Coordinating Committee’s 2016–2017 Strategic Plan [53]; and (v) type of funder (e.g., governmental, trust; see Table S2).

Details about participatory approaches included: (i) the types of community members involved (e.g., Autistic adults, parents of Autistic children); the breadth of community involvement (e.g., choosing research methods, recruiting participants, etc., following 23,24); and community members’ level of participation [21, 22]. Level of community participation was coded using an adapted version of Arnstein’s (1969) ladder of citizen participation, following the framework outlined in Tan, Crane, et al. (21; see Table 1). Studies that were framed as participatory but involved community members solely as research participants (e.g., responding to surveys or interviews) were coded as *informing*. Studies in which community members contributed to a single area of the research process (e.g., study design only) were coded as *consulting*. Studies in which community members were involved across some, but not all, areas of the research process were coded as *engaging.* Studies in which community members contributed across all areas of the research process were coded as *coproduced*. Where all areas of the research process were led by a team of mostly community members, studies were coded as *community-led*; where all areas were exclusively led by community members, studies were coded as *community-controlled*. Vague or unclear information about the study’s participatory approach was coded as ‘insufficient information’ (see Table S3). To facilitate data analysis and interpretation, we transformed our detailed coding schemes for types of community members and breadth of involvement into broader categories (see Tables S4 and S5).

### Quality appraisal

The methodological and reporting quality of all included articles was appraised by DT using the Mixed Methods Appraisal Tool (MMAT; [54]). This tool comprises several distinct sets of five criteria, with each set specifically designed to assess the methodological quality of either quantitative (e.g., “*Are the measurements appropriate?*”), qualitative (e.g., “*Are the findings adequately derived from the data?*”), or mixed methods (e.g., “*Are divergences and inconsistencies between quantitative and qualitative results adequately addressed?*”) research designs. Each criterion is rated “yes” if met, “no” if not met, and “can’t tell” if relevant information was not reported. The MMAT discourages appraisers from calculating an overall quality score or from excluding studies of low methodological quality. To facilitate interpretation, we used the following quality ratings: ‘good’ where all criteria had been met, ‘medium’ for most, but not all criteria, and ‘low’ where majority of criteria were unmet. Half of the articles (*n* = 191) were double coded by five volunteers with substantial agreement (76%).

### Community involvement statement

This review was collaboratively conceptualised by Autistic and non-autistic researchers and community members. Specifically, inputs from Autistic people were sought and implemented in the development of the review protocol (e.g., ensuring that the search strategy was comprehensive), the screening of titles, abstracts, and full texts, the development of the coding schemes, interpretation of review findings, and co-authorship. Nevertheless, due to time constraints and staff movement, Autistic people were not directly involved in the data extraction process. See Table S6 for an in-depth reflection of our participatory approach using the Guidance for Reporting Involvement of Patients and the Public-2 (GRIPP-2; 55).

## Results

### Overview of articles

Across the initial and updated searches, we identified 5,360 records through database searching. After removing duplicates, the titles and abstracts of 3,390 records were screened for eligibility. Of these, 1,605 full texts were reviewed, resulting in a final sample of 382 articles (Fig. 1).

### Quality of articles

Overall, 46% (*n* = 177) of the articles were found to be of ‘good’ quality, 45% (*n* = 172) of ‘medium’ quality, and 9% (*n* = 33) of ‘low’ quality (see Table 2). Common methodological issues include a lack of coherence between data sources, collection, analysis and interpretation in qualitative studies, a lack of sample representative of the target population in quantitative studies, and a lack of integration of qualitative and quantitative components in mixed methods studies.

**Table 2:** Quality of studies for each research method

Research Method*	No. of articles (%)	Quality		
		Low	Medium	Good
Qualitative	201 (53%)	16 (4%)	60 (16%)	125 (33%)
Quantitative descriptive	77 (20%)	10 (3%)	46 (12%)	21 (5%)
Quantitative randomised controlled trial	12 (3%)	0	10 (3%)	2 (0.5%)
Quantitative non-randomised trial	9 (2%)	1 (0.3%)	5 (1%)	3 (0.8%)
Mixed methods	25 (7%)	3 (0.8%)	18 (5%)	4 (1%)

Note. *15% (*n* = 58) of the studies adopted ‘multi-method’ designs, collecting both qualitative and quantitative data, but did not analyse the data using mixed methods. The quality of these studies was, therefore, assessed separately for each method

### Trend in participatory approaches

**Fig. 2:**
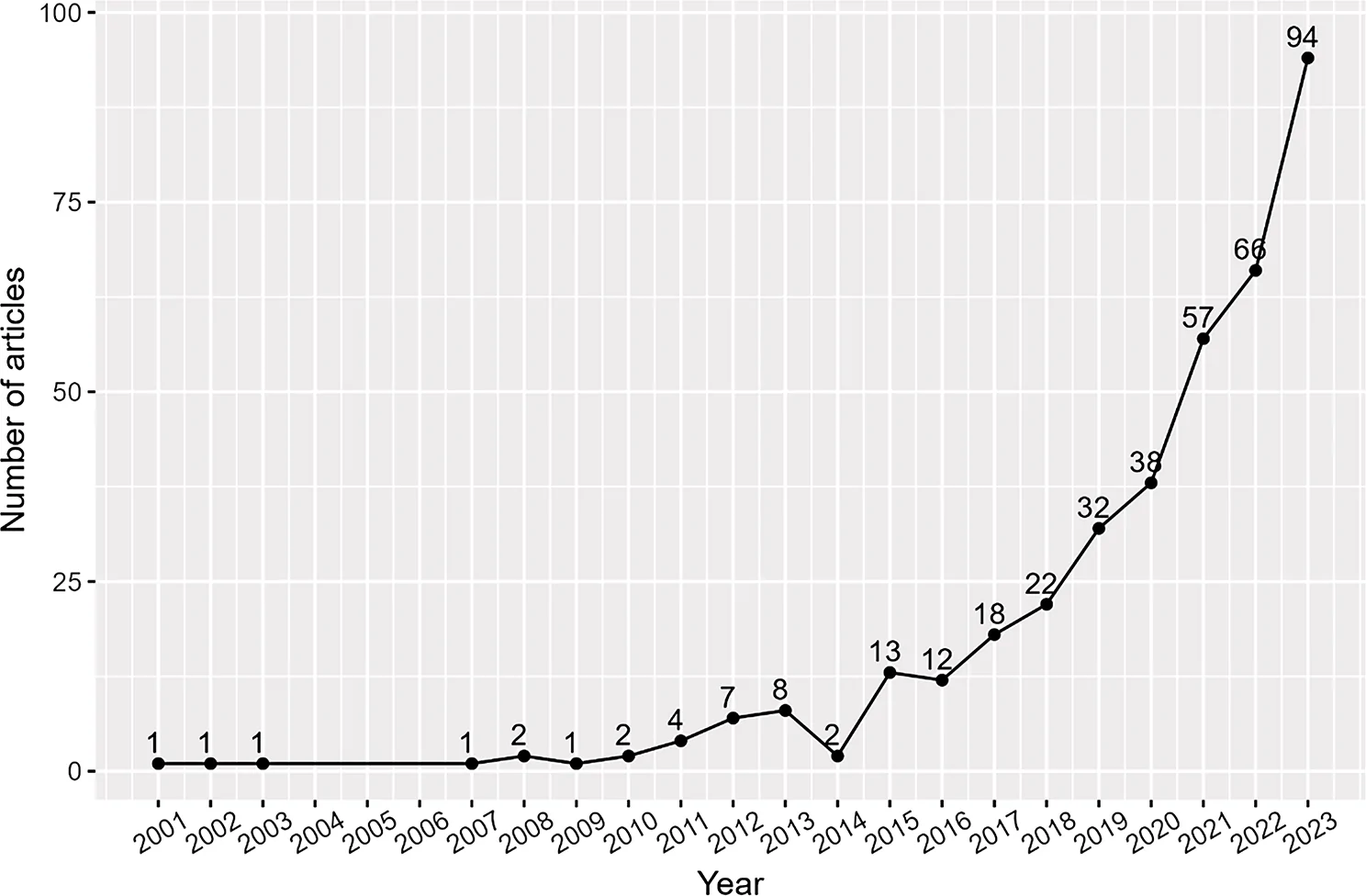
This line graph shows the number of articles reporting participatory approaches in autism studies between 2001 and 2023

From our search, it appears that the earliest paper reporting participatory approaches in the autism field was published in 2001. Over a 22-year period, there has been a sharp increase in the number of participatory autism studies, from the one study reported in 2001 to 94 studies in 2023, with nearly 95% (*n* = 362) of the included articles published in the decade between 2013 and 2023 (see Fig. 2 for a time-series trend).

### Community members involved

Across the 382 articles reviewed, two-thirds reported involving Autistic people (*n* = 256; 67%), with the majority involving Autistic adults (*n* = 184; 48%). While Autistic researchers were reported to be involved in a relatively small proportion of included studies (*n* = 68; 18%), it is possible that Autistic researchers were in some cases described simply as Autistic adults, thus making the distinction between lay Autistic adults and Autistic researchers unclear. In contrast, Autistic young people (*n* = 52; 14%) and Autistic people with complex support needs (*n* = 6; 0.02%) were much less frequently involved than Autistic adults and researchers. Other community members involved in research included professionals (*n* = 158; 41%) and families of Autistic people (*n* = 145; 38%; see Table 3).

**Table 3:** Community members involved in participatory autism research

Type of community members	No. of articles (%)
***Autistic People***	***256 (67%)***
Autistic young people	52 (14%)
Autistic adults	184 (48%)
Autistic people with complex support needs*	6 (0.02%)
Autistic researchers	68 (18%)
***Families of Autistic People***	***145 (38%)***
Parents of autistic young people	90 (24%)
Parents of autistic adults	21 (5%)
Parents of autistic people (unspecified age)	22 (6%)
Family members (unspecified connection)	16 (4%)
***Professionals***	***158 (41%)***
Clinicians/Healthcare providers	94 (25%)
Educators	72 (19%)
Professionals (unspecified speciality)	36 (9%)
***Administrators***	***26 (7%)***
Policymakers	5 (1%)
Funders	6 (2%)
Patient/Advocacy/Community organisations	20 (5%)
***Others***	***25 (7%)***
Community members (unspecified connection)	7 (2%)
Cannot be determined	18 (5%)

Note. Categories and their associated values in bold and italics are based on the broad coding scheme (see Table S4). The remaining sub-categories and their associated values (neither in bold nor italics) are based on the detailed coding scheme (see Table S3). As each study could involve more than one type of community member, percentages do not add up to 100%

*Includes Autistic people who are non-speaking, minimally-speaking, communicate in ways other than spoken language (e.g., using augmentative and alternative communication), have a co-occurring intellectual disability and/or require intensive support such as around-the-clock care or one-to-one assistance (see [55])

### Levels of participation

**Fig. 3:**
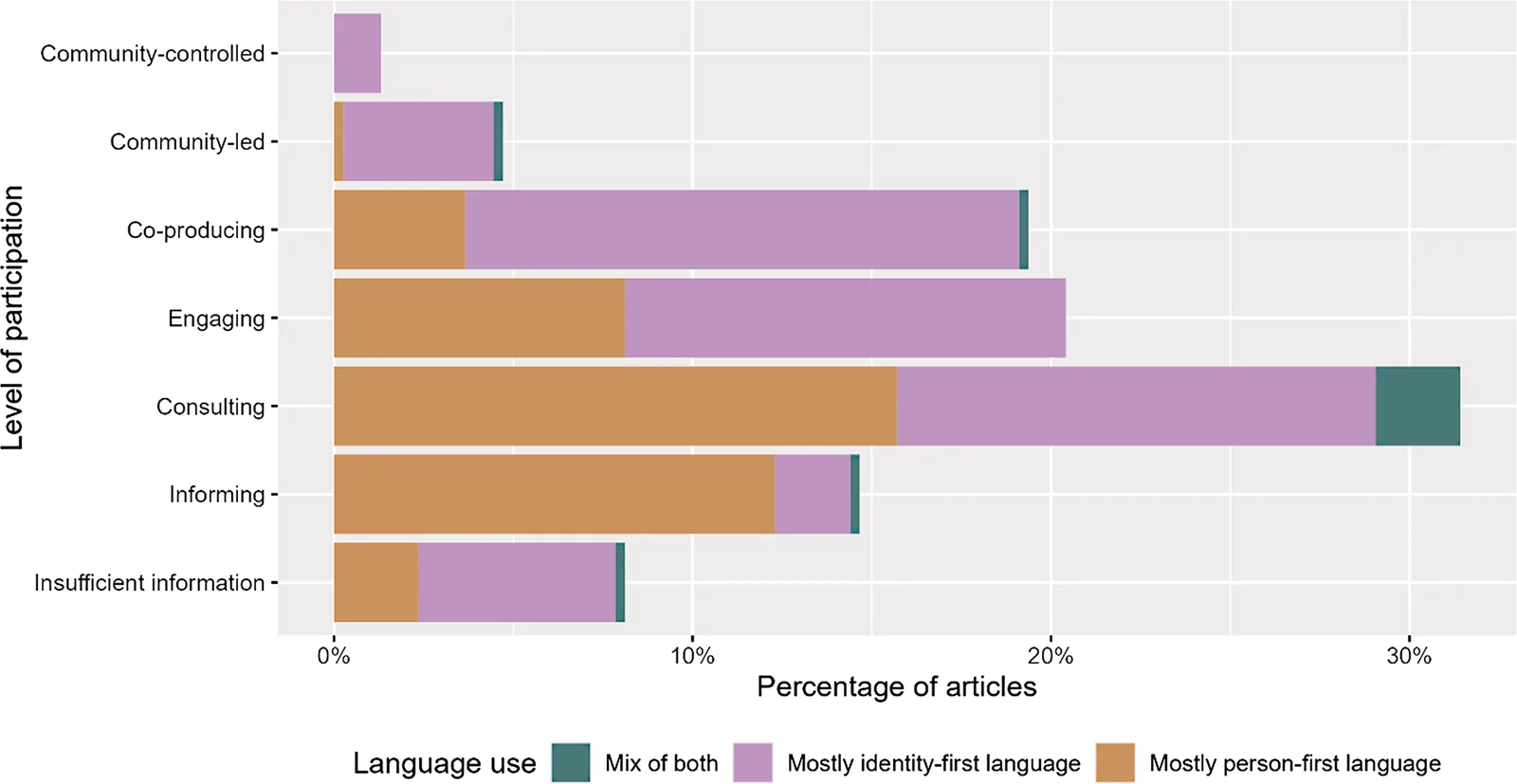
This bar chart shows the percentage of articles reporting participatory approaches across different levels of participation. Colours indicate the language used in these articles

Approximately one-third of the included studies involved community members in a *consulting* capacity (*n* = 120; 31%), followed by *engagement* (*n* = 78; 20%), and *co-production* (*n* = 74; 19%). Notably, despite being described as “participatory”, a substantial proportion of studies operated only at the *informing* level (*n* = 56; 15%). Many of these employed methods such as photovoice or photo-elicitation (*n* = 13; 3%) to facilitate more active forms of participation; however, community members were still primarily positioned as research participants who were informed about the study and contributed data, rather than as partners in decision making. A similar pattern was observed in co-design studies (*n* = 26; 7%), where community members contributed to co-designing a product or an intervention but rarely held substantive influence over how the studies were conceptualised or governed. Community-led (*n* = 18; 5%) and community-controlled (*n* = 5; 1%) levels of participation remain rare in autism research (Fig. 3).

**Fig. 4:**
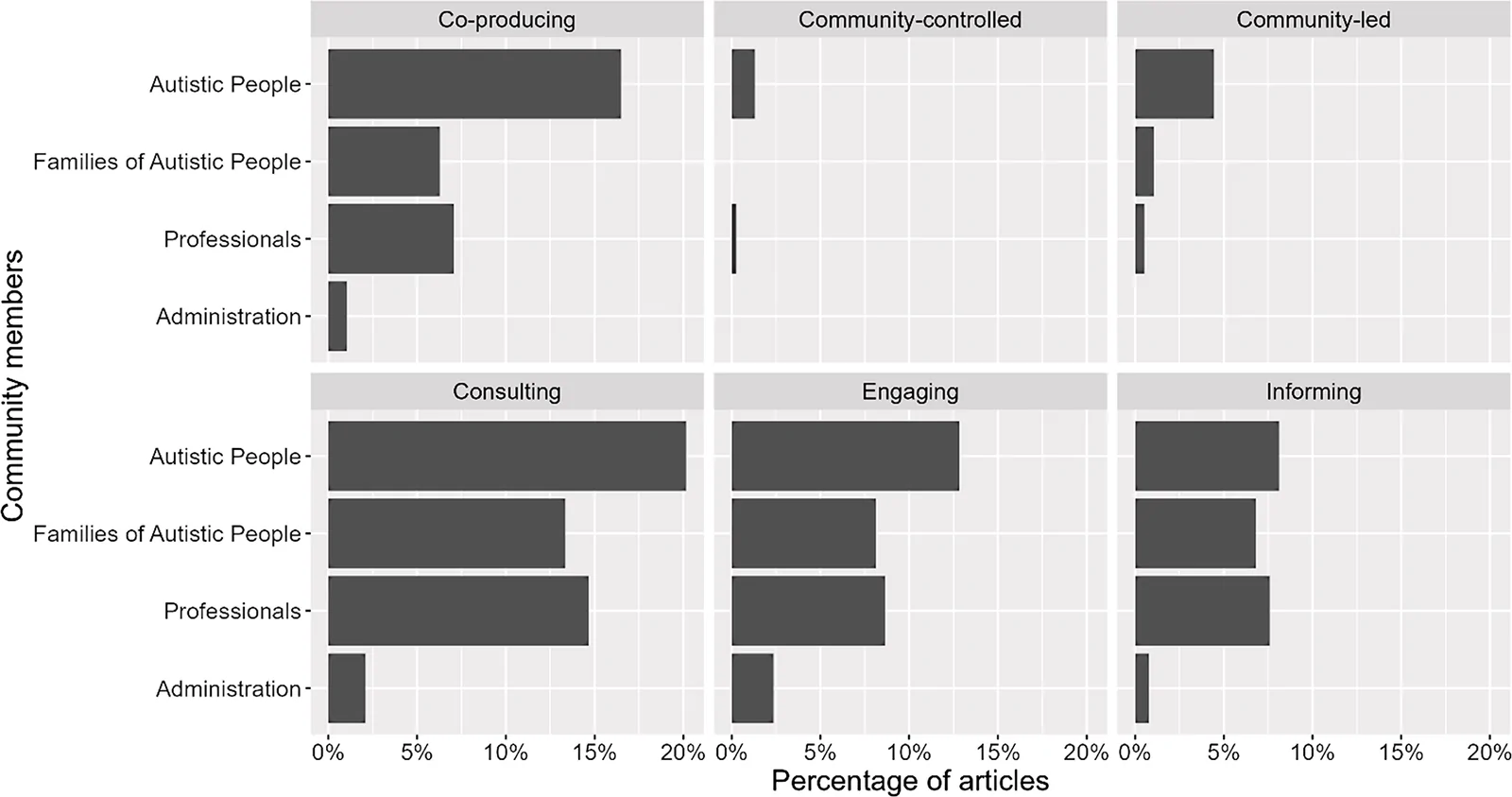
A panel of six bar charts showing the percentages of articles involving different groups of community members. Each chart represents each level of participation

We further analysed the types of community members involved across the six levels of participation (Fig. 4). Autistic people were most often consulted on studies (*n* = 77; 20%), followed by co-producing (*n* = 63; 16%). Professionals (e.g., educators and clinicians) were also often consulted (*n* = 56; 15%), followed by families of Autistic people (*n* = 51; 13%). See Table S7 for a detailed breakdown of frequencies and percentages across different groups of community members and levels of participation.

### Breadth of participation

In terms of the type of research activity, most studies reported involving community members in study design (*n* = 284; 74%), followed by analysis (*n* = 126; 33%), and dissemination (*n* = 111; 29%; see Table 4). Based on the detailed coding scheme, it appears that community members were most involved in designing/modifying interview schedules and/or survey questions (*n* = 179; 47%) and least involved in grant proposal writing (*n* = 15; 4%) and presenting at meetings or conferences (*n* = 15; 4%).

**Table 4:** Breadth of community involvement across various research activities

Type of research activity	No. of articles (%)
***Conceptualisation***	***105 (27%)***
Developing community-based theories	44 (12%)
Grant proposal writing	15 (4%)
Background research	87 (23%)
***Design***	***284 (74%)***
Choosing research method	118 (31%)
Developing sampling procedures	75 (20%)
Designing/Modifying interview schedules and/or survey questions	179 (47%)
Providing inputs on public-facing materials	36 (9%)
Developing/Adapting intervention	69 (18%)
***Implementation***	***96 (25%)***
Recruiting study participants	67 (18%)
Implementing intervention	23 (6%)
Collecting primary data	51 (13%)
***Analysis***	***126 (33%)***
Analysing data	94 (25%)
Interpreting study findings	117 (31%)
***Dissemination***	***111 (29%)***
Writing reports/journal articles	106 (28%)
Giving presentations at meetings/conferences	15 (4%)
***Insufficient information to determine***	***21 (5%)***

Note. Categories and their associated values in bold and italics are based on the broad coding scheme (see Table S5). The remaining sub-categories and their associated values (neither in bold nor italics) are based on the detailed coding scheme (see Tables S3 and S5). As each study could involve community members in more than one type of activity, percentages might add up to more than 100%

### Factors associated with participatory approaches

We examined four factors that may be associated with the adoption or reporting of participatory approaches in the autism field, including (i) country, (ii) research areas, (iii) research methods, and (iv) funders.

#### Country

**Fig. 5:**
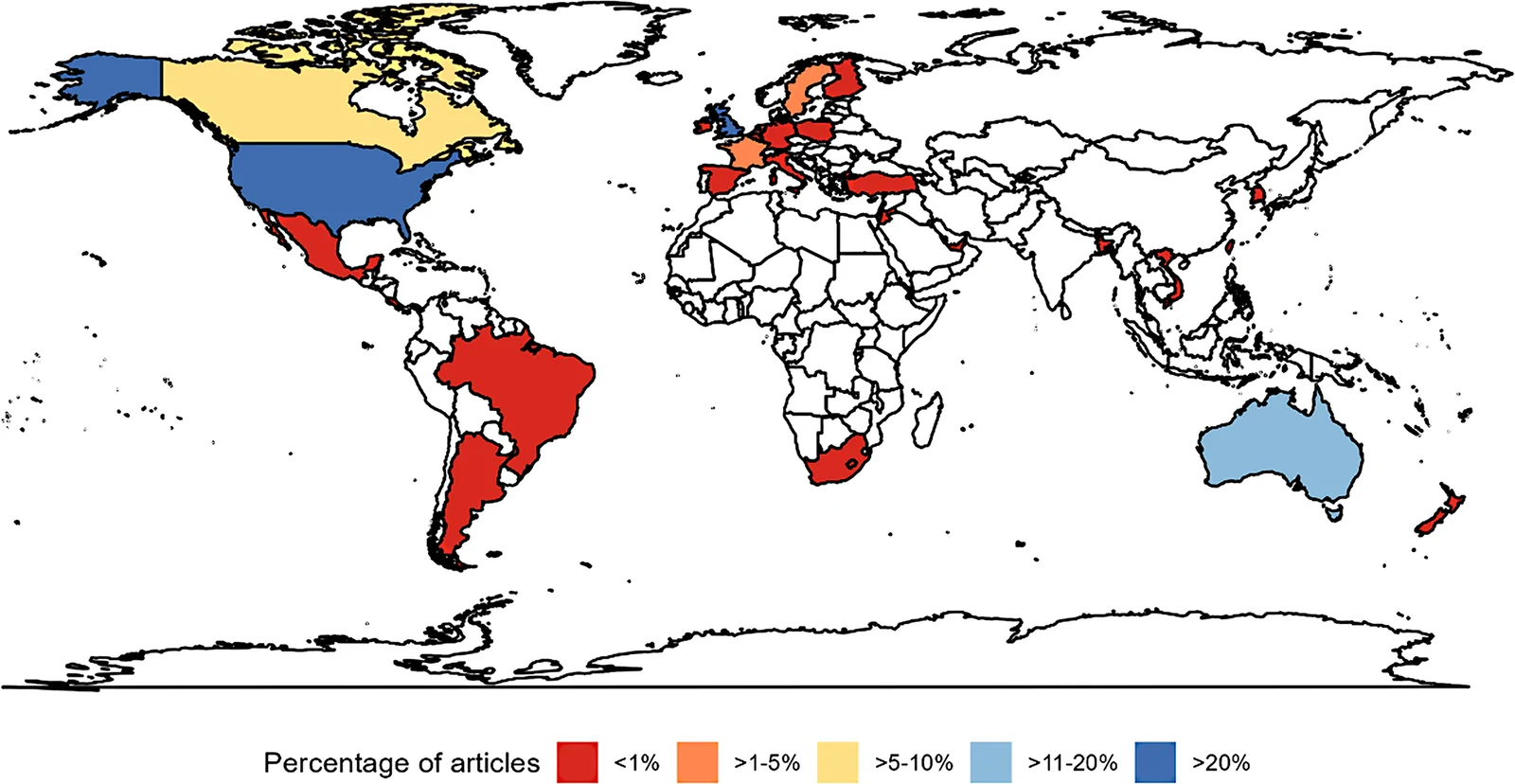
Geographical distribution of participatory autism research across the globe. The percentage of studies included in this review originating from each country is shown according to the scale above, ranging from less than 1% to over 20%. and percentages across different countries grouped by levels of participation

A considerable majority (86%) of participatory autism research originated from four countries: the USA (*n* = 124; 32%), UK (*n* = 122; 32%), Australia (*n* = 52; 14%) and Canada (*n* = 31; 8%; see Fig. 5). An additional 25 countries were represented in our analysis, each having produced between 1 and 4 relevant publications. See Table S8 for a detailed breakdown of frequencies.

#### Research topics

Overall, participatory approaches were most common in applied research, including research investigating lifespan issues (*n* = 167; 44%), interventions (*n* = 98; 25%), and services and supports (*n* = 66; 17%; see Table 5). In contrast, none of the included studies were related to genetic and environmental factors and very few focused on biology (*n* = 16; 4%). Among studies focusing on lifespan issues, about one-third were co-produced with community members (*n* = 48; 29%), one-third involved community members in consulting roles (*n* = 46; 28%), and 19% in engagement (*n* = 31).

#### Research methods

Over half of the included studies employed qualitative methods (*n* = 201; 53%), while 98 studies (26%) adopted quantitative methods (including descriptive studies and non-randomised controlled trials, and randomised controlled trials) and 83 (22%) studies used mixed methods (Table 2). See Table S9 for a detailed breakdown of frequencies and percentages across different research methods grouped by levels of participation.

#### Funders

Most participatory autism research was funded by governmental bodies (*n* = 167; 44%) or went unfunded (*n* = 122; 32%; Table 6). Among studies that received governmental funding, 31% (*n* = 51) consulted with, 24% (*n* = 40) engaged with, and 23% (*n* = 39) co-produced with community members. Notably, all the community-controlled studies were unfunded (*n* = 5; 100%).

### Impact of participatory approaches

During the coding process, the research team observed certain patterns of language use (identity-first, person-first, or combination of both) and the journals in which participatory autism studies were published. We therefore conducted further subsidiary and unplanned coding and analyses to examine the potential impact of adopting participatory approaches. Specifically, we examined if participatory approaches were associated with language use and the journals in which articles were published.

**Table 5:** Research topics that adopted participatory approaches, grouped by levels of participation

Research topics	*N* (%)^†^	Levels of participation No. of articles (%)^‡^						
		Informing	Consulting	Engaging	Coproducing	Community Led	Community Controlled	Insufficient information
Screening and diagnosis	13 (3%)	1 (8%)	8 (62%)	2 (14%)	1 (8%)	0	0	1 (8%)
Biology^*^	16 (4%)	3 (19%)	5 (31%)	4 (25%)	2 (13%)	0	0	2 (13%)
Genetic and environmental factors	0	0	0	0	0	0	0	0
Interventions	98 (25%)	23 (23%)	35 (36%)	26 (27%)	8 (8%)	0	0	6 (6%)
Services and supports	66 (17%)	11 (17%)	22 (33%)	13 (20%)	11 (17%)	1 (2%)	0	8 (12%)
Lifespan	167 (44%)	13 (8%)	46 (28%)	31 (19%)	48 (29%)	13 (8%)	4 (2%)	12 (7%)
Infrastructure and prevalence	22 (6%)	5 (23%)	4 (18%)	2 (9%)	4 (18%)	4 (18%)	1 (5%)	2 (9%)

Note. ^*^Includes topics on brain structure and function and cognitive studies [53]

^†^Percentage out of the 382 included articles

^‡^Percentage out of the total number of articles on each research topic that were included in this review (i.e., *N*)

**Table 6:** Participatory research funding sources, grouped by levels of participation

Funding source	*N* (%)^*^	Levels of participation No. of articles (%)^†^						
		Informing	Consulting	Engaging	Coproducing	Community Led	Community Controlled	Insufficient information
Governmental	167 (44%)	17 (10%)	51 (31%)	40 (24%)	39 (23%)	10 (6%)	0	10 (6%)
Industry	5 (1%)	1 (20%)	1 (20%)	2 (40%)	1 (20%)	0	0	0
Service providers	6 (2%)	0	3 (50%)	1 (17%)	1 (17%)	0	0	1 (16%)
Trust	104 (27%)	16 (15%)	28 (27%)	23 (22%)	23 (22%)	4 (4%)	0	10 (10%)
University	78 (20%)	15 (19%)	15 (19%)	18 (23%)	17 (22%)	7 (9%)	0	6 (8%)
None	122 (32%)	21 (17%)	47 (39%)	19 (16%)	15 (12%)	3 (2%)	5 (4%)	12 (10%)

Note. ^*^Percentage out of the 382 included articles. As each study could be funded by more than one type of funder, percentages therefore do not add up to 100%

^†^Percentage out of the total number of articles funded by each funding source (i.e., *N*)

#### Language use

Of the included studies, more than half adopted identify-first language (*n* = 207; 54%) in their reporting, followed by person-first language (*n* = 162; 42%) and a mix of both (*n* = 13; 4%). A greater proportion of studies used identity-first language when they operated on the higher rungs of the participatory ladder (co-producing: 80%; community led: 89%; community controlled: 100%). Conversely, more person-first language was used on the lower rungs (informing: 84%; consulting: 50%; see Fig. 3). See Table S10 for a detailed breakdown of frequencies and percentages across different language use grouped by levels of participation.

#### Journals

Studies included in this review were published across 125 journals, with just under half (*n* = 187; 49%) drawn from the 11 journals specialising in autism research. Studies published across these specialist journals were examined in more detail (see Table 7). The journal *Autism* showed the highest concentration of participatory autism research (*n* = 106; 28%), followed by *Journal of Autism and Developmental Disorders* (*n* = 41; 11%) and *Autism in Adulthood* (*n* = 19; 5%). Over half of the participatory studies published in *Autism* worked with community members either in consulting (*n* = 29; 27%) or more substantive co-producing (*n* = 37; 35%) roles. In contrast, almost half of the participatory studies published in the *Journal of Autism and Developmental Disorders* consulted with the community (*n* = 19; 46%). Notably, of the few studies reporting participatory autism research in *Autism in Adulthood*, all operated on the higher rungs of the participatory ladder (co-producing: *n* = 6; 32%; community led: *n* = 4; 21%; community controlled: *n* = 1; 5%).

**Table 7:** Autism-specific journals in which participatory autism studies were published, grouped by levels of participation

Journal names	*N* (%)^*^	Levels of participation No. of articles (%)^†^						
		Informing	Consulting	Engaging	Coproducing	Community Led	Community Controlled	Insufficient information
*Autism*	106 (28%)	5 (5%)	29 (27%)	16 (15%)	37 (35%)	5 (5%)	1 (1%)	13 (12%)
*Journal of Autism and Developmental Disorders*	41 (11%)	4 (10%)	19 (46%)	6 (15%)	8 (20%)	0	1 (2%)	3 (7%)
*Autism in Adulthood*	19 (5%)	0	0	8 (42%)	6 (32%)	4 (21%)	1 (5%)	0
*Autism Research*	9 (2%)	1 (11%)	5 (56%)	2 (22%)	0	0	0	1 (11%)
*Autism & Developmental Language Impairments*	4 (1%)	0	1 (25%)	2 (50%)	0	0	0	1 (25%)
*Research in Autism Spectrum Disorders* ^*‡*^	3 (0.8%)	0	1 (33%)	1 (33%)	1 (34%)	0	0	0
*Molecular Autism*	1 (0.3%)	0	0	1 (100%)	0	0	0	0
*Advances in Autism*	1 (0.3%)	1 (100%)	0	0	0	0	0	0
*Autism Policy & Practice*	1 (0.3%)	1 (100%)	0	0	0	0	0	0
*Autism Research and Treatment*	1 (0.3%)	0	1 (100%)	0	0	0	0	0
*Focus on Autism and other Developmental Disabilities*	1 (0.3%)	0	0	1 (100%)	0	0	0	0

Note. ^*^Percentage out of the 382 included articles

^†^Percentage out of the total number of articles published in each journal (i.e., *N*); ^‡^Now known as *Research in Autism*

## Discussion

This mapping review examined the extent and nature of participatory approaches within autism research by analysing 382 studies published between 2001 and 2023 that reported community involvement. Our findings reveal a field experiencing rapid growth in participatory rhetoric but falling short of meaningful power-sharing in practice. In what follows, we discuss these patterns and consider their implications.

### The growth of participatory rhetoric in autism research

The overall increase in reporting participatory approaches is encouraging, particularly in the last decade. Our review identified just one study reporting community involvement in 2001, compared to 94 in 2023, with 95% of all included studies (*n* = 362) published between 2013 and 2023.

Several factors likely contributed to this increase. First, social media enabled Autistic advocates to publicly critique autism research and organise collective action. Hashtags such as #NothingAboutUsWithoutUs and #ActuallyAutistic created organised pressure that amplified community voices and facilitated rapid mobilisation around harmful research practices – as demonstrated by responses to initiatives like Spectrum 10 K [14, 30, 56–59]. Second, growing recognition that much of autism research misaligned with community priorities [10, 60] prompted calls to involve Autistic people in decision-making about research agendas and processes. Third, the rise of the neurodiversity paradigm, which centres Autistic people’s experiential expertise and challenges deficit-focused frameworks, provided conceptual grounding for participatory approaches [61, 62].

The particularly sharp increase from 32 studies in 2019 to 94 in 2023 – nearly tripling in just five years – coincides with two developments: *Autism*’s 2021 introduction of a policy mandating the reporting of community involvement (discussed below; 23), and the COVID-19 pandemic. The pandemic-driven shift to remote work normalised virtual engagement, making participation more accessible for many Autistic people by reducing barriers related to travel, unfamiliar environments, and sensory demands, while enabling greater control over participation conditions [63] – though remote arrangements may disadvantage those without infrastructure access [64].

Nevertheless, the quantitative trend toward increased reporting of community engagement must be interpreted with caution. A cursory search of PsycINFO identified 63,875 autism-related articles published during this period, meaning that studies adopting participatory approaches only represent approximately 0.6% of all published autism research. The increase may reflect genuine shifts in research practice, but also increased visibility due to journal policies or strategic framing whereby researchers highlight minimal consultation to appear participatory [21]. Most critically, an increase in the *reporting* of participation does not necessarily translate into an increase in the *quality* or *depth* of participation. As the following sections demonstrate, while participatory language has become increasingly normative, genuine power-sharing and community control remain rare.

### The characteristics of participatory autism research

#### Who is involved?

Two-thirds of the included studies (67%) reported involving Autistic people. While this represents an encouraging majority, this nonetheless means that one-third of research claiming to be “participatory” excluded Autistic people entirely, raising fundamental questions about what constitutes legitimate participation in autism research when the ultimate beneficiaries are absent. Even when Autistic people were involved, representation was uneven: Autistic adults contributed to nearly half of all studies (48%), a participation rate that is roughly comparable to the proportion of studies focused on adult-specific issues (44%). This picture was markedly different for Autistic young people: although research focused on this population was considerably more common, with 42% of studies addressing interventions and services for young people, Autistic young people contributed to only 14% of the included studies. This is consistent with a recent review which identified just five studies that adopted participatory approaches with Autistic young people in education studies [43]. This gap suggests that, unlike for adults, growing research attention to young people’s needs [65] has not been matched by their inclusion as collaborators. Strikingly, less than 1% of the studies involved Autistic people with complex support needs as co-researchers. While these disparities may reflect practical and ethical challenges in engaging young people [66, 67] and those with complex support needs [55], it risks privileging adult perspectives and/or the views of those with fluctuating or “less complex” support needs.

A substantial proportion of studies involved families of Autistic people (38%) and/or professionals (41%). While these stakeholders can play valuable supportive roles, their involvement alongside – or instead of – Autistic people warrant careful consideration. Families and professionals often have different, and sometimes conflicting, priorities and perspectives compared to Autistic people [10]. When these divergent perspectives are given equal or greater weight, research agendas risk being shaped more by caregiver burden [68] or professional concerns than by Autistic priorities. The principle of “nothing about us without us” suggests that Autistic people should hold primary epistemic authority in research about them, with other stakeholders occupying supporting rather than central roles.

Autistic insider-researchers were reported as contributing to only 18% of studies, though – as noted above – this may underestimate actual involvement. Autistic researchers’ presence is significant, bringing both lived experience and academic expertise. However, the Southern participatory research tradition’s emphasis on addressing power differentials [19, 20] highlights an important tension: while Autistic researchers are marginalised within academia [34, 69, 70], they nevertheless occupy positions of relative power compared to non-academic Autistic community members [21, 71]. As such, Autistic researchers should neither bear sole responsibility for making autism research participatory nor substitute for non-academic Autistic community members [72].

At the same time, Autistic people are not a monolithic group, and particular attention must be paid to intersectional marginalisation among both Autistic insider-researchers and non-academic Autistic community members alike. Meaningful participation requires involving diverse Autistic people, including but not limited to those who are non-speaking, who have co-occurring intellectual disability, who are racially marginalised, who are gender minorities, and those outside academia who are often excluded from traditional knowledge production [55].

#### The levels of participation

Community involvement in autism research was concentrated at the lower rungs of the participatory ladder, with academic researchers retaining primary decision-making power. Participation was most commonly at the consulting (31%) and engaging (20%) levels, representing tokenistic involvement with academics holding most power [21]. Notably, 15% of studies operated at the informing level, with community members contributing data – often through collaborative methods, like photovoice – but holding little influence over how the research was conceptualised. Community-led research represented just 5% of studies; and community-controlled research, where community members hold ultimate decision-making authority, accounted for only 1% of the sample. Even amongst studies involving Autistic people specifically, consultation remained most common (20%), followed by co-production (16%). Community-led (5%) and community-controlled (1%) research remained rare, suggesting that even when Autistic people are involved, they are typically not granted substantive decision-making power.

This pattern reveals an important distinction between participation as method and participation as governance or epistemic authority. Some studies employed methods, such as photovoice or co-design, that are purportedly participatory or community responsive, but where decisions about research questions, interpretation, authorship, and dissemination are still controlled by researchers. In these cases, participatory methods were, then, used in research processes that remained fundamentally researcher-governed and operating at informing, consulting and engaging levels. In contrast, co-producing, community-led, and community-controlled approaches reflect participation as governance and epistemic authority, wherein community members hold equal or substantive influence over decision-making and over what counts as legitimate knowledge.

This distinction maps onto the two traditions introduced earlier – participation as method aligns more closely with the Northern tradition’s emphasis on practical, collaborative problem-solving, whereas participation as governance and epistemic authority aligns with the Southern tradition’s emphasis on shifting decision-making power to marginalised communities. Our findings suggest that much of what is currently labelled “participatory” in autism research reflects the former – methodological inclusion – rather than the latter, even though the motivations articulated by Autistic advocates and the Southern tradition alike call explicitly for the redistribution of power in knowledge production [19, 20], not merely the adoption of more inclusive methods.

#### Breadth of participation

Community involvement was concentrated in study design (73%), with substantially less engagement in analysis (33%) and dissemination (29%). Within these stages, community members were most involved in designing interview schedules or survey questions (47%) but rarely in grant proposal writing (4%) or presenting at conferences (4%).

This pattern suggests that community involvement often occurs in the “safer” stages that do not fundamentally challenge academic control over knowledge production. While involving community members in refining materials can improve research quality, it does not redistribute epistemic authority. Stages where fundamental decisions are made – what questions are worth asking, how data should be interpreted, how findings should be communicated – remain largely under academic control. Limited involvement in analysis is particularly significant, as this is where raw data becomes knowledge. When community members are excluded, academics retain power over what data *means*, reproducing traditional research-participant hierarchies even within ostensibly participatory frameworks. The concentration of involvement in design also raises questions about timing. If community members are only consulted *after* research questions and frameworks are established – as is often the case when involvement is limited to “refining” materials – their influence is constrained from the outset; therefore, what is meaningful and important to Autistic people still missed.

In coding the included articles, we encountered a proportion of studies that provided insufficient or vague information to reliably code key dimensions of participation. Eight (2%) articles were unclear in terms of the community members involved, 31 (8%) could not be coded for level of participation, and 17 (4%) were vague on how community members were involved. This gap in reporting is important to highlight, as current guidelines (e.g., GRIPP-2) [73, 74] recommend that studies clearly report who community members were, how they were involved, which aspects of the research their involvement shaped, the impact of that involvement, and reflections on the enablers and barriers to community involvement, along with recommendations for improving practice in future [21].

While such vague reporting on participatory approaches may reflect a lack of familiarity with good reporting standards, it may also stem from structural issues, such as journal word limits and disciplinary norms, which restrict how much space authors can devote to describing participatory processes in detail. These are, however, not insurmountable barriers. Some journals have committed to additional word allowances specifically for reporting participatory processes (e.g., *Autism*, 39). Most journals also allow supplementary materials, as we have done in the current review (see Table S6), and some research teams have chosen to report their participatory processes in dedicated, standalone articles [2, 75]. Taken together, these examples suggest that space constraints need not preclude comprehensive reporting of participatory processes, and we encourage researchers and journals alike to adopt such strategies more widely.

### Factors associated with participatory approaches

We examined four factors that may be associated with participatory approaches. First, participatory autism research is heavily concentrated in four Western nations (USA, UK, Australia, and Canada), which accounted for 86% of all participatory studies. This concentration reflects broader patterns in autism research, which is conducted predominantly in North America and Europe [76, 77]. However, the geographic skew in participatory research also replicates colonial dynamics in knowledge production [78, 79], risking the universalisation of Western-centric frameworks and marginalising non-Western understandings of autism and context-specific priorities [79–81]. Moreover, this inequity means that the benefits of participatory research accrue primarily to Autistic people in wealthy, English-speaking nations, while Autistic people elsewhere remain largely under-represented.

We nevertheless acknowledge that the current review was limited to English-language, peer-reviewed publications indexed in primarily Western databases, thereby excluding dissertations, reports, community publications, and other grey literature. Given that our review is fundamentally concerned with where power sits in knowledge production, this exclusion may have systematically omitted community-led and community-controlled work, particularly those produced outside academic institutions and Western publishing systems. More broadly, this raises the question of whether peer-review academic publishing itself privileges academic-led knowledge production, potentially rendering the very forms of participation most aligned with emancipatory, power-sharing ideals least visible within the current review.

Second, participatory approaches were most often adopted in applied research, particularly studies examining lifespan issues, interventions, and services and supports. In stark contrast, no participatory study focused on genetic and environmental factors, and very few addressed biological aspects of autism. This distribution broadly mirrors studies asking Autistic people what they want from autism research which have consistently found genetic and biological research ranked among the lowest priorities while services, intervention, and lifespan-related concerns rank highest [10, 60]. However, this pattern may also reflect researchers’ own assumptions that community input is not well suited to genetic and biological research [25], despite the profound social and ethical implications of basic science [13]. In sum, most funded autism research focuses on genetic and biological questions [53, 82] that are not community priorities [60] and proceed without Autistic input [21, 40], despite these topics being where input may be most ethically imperative [13].

We are also cautious about the assumption that certain study designs are inherently better suited to participatory approaches than others [25]. Beyond autism research, fields such as agriculture and environmental science have demonstrated that even highly technical, traditionally researcher-led studies can meaningfully incorporate experiential expertise [83, 84]. In our view, all types of research designs can – and should – enact participatory approaches, though more traditional or technically complex designs may require greater effort to depart from conventional, researcher-controlled ways of working.

Third, over half employed qualitative methods, likely reflecting qualitative research’s emphasis on lived experiences and the mistaken assumption that participatory research must be qualitative [16]. This may be compounded by the fact that positionality statements – which often report how researchers’ lived experiences and values shape their studies – are commonly expected in qualitative studies [85, 86], potentially inflating the apparent proportion of qualitative studies identified as participatory in this review. Importantly, however, quantitative evidence often carries greater weight in policy and practice decisions [87]. It is therefore imperative that community influence be extended to other research designs, including quantitative and mixed-method approaches.

Fourth, most participatory autism research was government-funded (44%) or unfunded (32%). Government funding was associated with higher levels of participation, with 24% involving engagement, 23% co-production, and 6% community leadership. However, all five community-controlled studies were unfunded. While some government agencies have introduced requirements or incentives for community involvement such as the Patient-Centered Outcomes Research Institute in the U.S., U.K. Research and Innovation, and Australia’s National Health and Medical Research Council [88], the fact that all community-controlled research was unfunded raises important questions about whether traditional funding structures including eligibility criteria (e.g., institutional affiliations) are compatible with truly community-controlled research.

Existing funding structures may also help explain why community involvement was less common in study conceptualisation and dissemination, as reported earlier. Without dedicated funding, researchers may lack the resources to meaningfully engage community members from the outset of the project, particularly in writing grant applications, when priorities and research questions are first established. Furthermore, even when funding is secured, project timelines are often insufficient to sustain community involvement through to dissemination, which frequently occurs well beyond the official funding period given the lengthy publication process. As such, current funding mechanisms may inadvertently constrain community involvement to the middle stages of the research process.

### Impact of participatory approaches

To examine potential impacts of adopting participatory approaches, we conducted subsidiary analyses examining language use and publication patterns. These analyses reveal that participation depth is associated with both how Autistic people are described and where research is published.

Over half of included studies (54%) used predominantly identity-first language (e.g., “Autistic people”), while 42% used person-first language (e.g., “people with autism”). Strikingly, language use appears to be associated with participation level. Studies operating at higher rungs tended to use identity-first language (co-producing: 80%; community-led: 89%; community-controlled: 100%), while those at lower levels tended to use person-first language (informing: 84%; consulting: 50%). This pattern is significant because identity-first language which views autism as integral to identity [89] is preferred by many Autistic people [90, 91]. The association between deeper participation and identity-first language suggests that meaningful community involvement may influence both the research processes and the conceptual frameworks researchers adopt. When Autistic people hold greater decision-making power, research appears more likely to reflect community language preferences.

Studies were published across 125 journals, with notable differences in participation depth among autism-specialised journals. *Autism* published the most participatory research (28%), with studies mostly operating at consulting (27%) or co-producing (35%) levels. *Journal of Autism and Developmental Disorders* (11% of included studies) concentrated at consulting levels (46%). Most strikingly, all participatory studies in *Autism in Adulthood* (5% of included studies) operated at higher participation rungs (co-producing: 32%; community-led: 21%; community-controlled: 5%). These patterns likely reflect journal positioning and policies. *Autism in Adulthood*, established in 2019 with explicit commitments to centring Autistic voices, appears to attract or select for deeper community involvement. *Autism*’s 2021 community involvement reporting policy increased visibility but not necessarily depth [21]. *Journal of Autism and Developmental Disorders*’ concentration at consulting levels may reflect the journal’s traditional positioning within developmental psychology and psychiatry, fields historically less engaged with participatory approaches. These findings suggest journal policies and editorial priorities can shape how deeply participation is enacted, though even in progressive journals, community-led and community-controlled research remains rare.

### Strengths and limitations

This review has several strengths. First, with 382 included studies, it represents the largest and most comprehensive systematic examination of participatory autism research to date. Second, our pre-registered protocol and systematic search across multiple databases provide broad coverage of the field. Third, by examining multiple dimensions of participation – who is involved, at what level, in which activities, and under what conditions – we provided a nuanced account of participatory activities in autism research.

Nevertheless, there are also several limitations. First, we necessarily relied on what authors reported in published articles that used terms like “participatory”, “community involvement” and related keywords, thus potentially missing studies that involved community members without explicitly labelling their approach as such. Relatedly, our coding system, which treats involvement across more research stages as an indicator of level or depth of participation, may not adequately capture the depth or breadth of *influence.* Community members may be involved across many stages in relatively superficial ways, or they may hold substantial influence over a small number of critical stages, such as setting the research question or interpreting findings. As we were only able to code what was reported in published articles – and such reporting was often insufficiently detailed to distinguish these possibilities – our coding system may consequently over-value broad but shallow involvement or under-value focused but meaningful influence. Future research may consider adopting frameworks such as Evaluating Levels of Participation in Autism Research Tool [92] which distils participation at each stage of the research process in greater granularity, or the Public and Patient Engagement Evaluation Tool [93] that evaluates the influence and impact of community members on research projects, thus offering a more precise approach than was feasible within the scope of the current review.

Second, as reported in our variations from the registered protocol (see Method), we were unable to evaluate the quality of *reporting* of participatory approaches. While we assessed methodological quality using the MMAT [54], as informed by the PRISMA guidelines for systematic reviews [47], methodological reporting quality is distinct from the quality of participatory reporting; a study may be methodologically well-reported yet provided limited detail on participatory approaches, or may report deep community involvement yet lack sufficient methodological detail for quality appraisal. Future studies should therefore consider evaluating the quality of reporting participatory approaches using existing tools such as GRIPP-2 [73].

Third, we did not code for whether community members were compensated for their time. While compensation is an important marker of respect for community members, we emphasise that compensation alone represents a baseline expectation of ethical practice rather than direct evidence of power-sharing. We also did not capture information regarding the demographic characteristics of community members, such as race, gender, social class, or educational background, which are critical to understanding the intersectional marginalisation discussed earlier. We recommend that future studies explicitly attend to both compensation practices and the demographic diversity of community members to enable a more thorough evaluation of participatory practice.

## Conclusions

This systematic review of 382 studies reveals a field experiencing rapid growth in participatory rhetoric but limited progress in redistributing power. While participatory language has become increasingly normative in autism research, most studies operate at consultative levels, with community-led and community-controlled research remaining rare. Community involvement concentrates in research stages like study design that do not fundamentally challenge academic control over knowledge production, with substantially less involvement in analysis or dissemination. Moreover, participatory research is unevenly distributed – concentrated in applied research areas, qualitative studies, and Western countries – suggesting structural barriers shape where and how participation occurs. Moving beyond consultative practices toward the emancipatory ideals articulated by Autistic advocates would require systemic changes. These include funding structures that resource deeper participation across the full research lifecycle, particularly the currently under-resourced stages of conceptualisation and dissemination; institutional policies recognising community expertise as legitimate expertise; deliberate efforts to include Autistic people who remain underrepresented in current research including young people and those with complex support needs; and willingness among researchers to share decision-making authority over research concerning Autistic peoples’ lives.

## Supplementary Information

Below is the link to the electronic supplementary material.

Supplementary Material 1

## Data Availability

All data, analysis codes written in RStudio [44], research materials (including our coding scheme), an interactive dashboard, and preregistered review protocol are available at https://osf.io/aw4km/overview?view_only=84b23759419a4b36864a3f4c8b9f70b6.

## Footnotes

1 We did not code for disciplinary background of research teams, as this information was not consistently reported and, where present, was typically embedded within positionality statements of qualitative studies. We instead coded the *research topic* addressed by each study, which reflects the focus of the research rather than the disciplinary affiliation of the team.

## Abbreviations

BMJ: British Medical Journal
PPI: Patient and Public Involvement
PRISMA: Preferred Reporting Items for Systematic reviews and Meta-Analyses
MMAT: Mixed Methods Appraisal Tool
GRIPP-2: Guidance for Reporting Involvement of Patients and the Public (Version 2)
