# Supplementary material for: A comprehensive mapping review of participatory autism research over a 22-year period
Source: Res Involv Engagem. 2026 Nov 2;12:179. doi: 10.1186/s40900-026-00998-y (PMC13726076; doi:10.1186/s40900-026-00998-y)
Supplement: Supplementary file 1 [file 40900_2026_998_MOESM1_ESM.docx]

Supplementary File

Table S1

*Search Strategy*

|  | **Autism** | **Participatory Research** | **Community Involvement** |
| --- | --- | --- | --- |
| **CONCEPTS AND SYNONYMS** | - Autism spectrum disorder - Autism spectrum condition - Autistic disorder - Asperger’s - High functioning autism - Pervasive Developmental Disorder-Not Otherwise Classified | - Community-based Participatory Research - Action Research - Inclusive Research - Community-led Research - Community-informed Research - Co-Production Research - Co-Produced Research - Co-Designed Research - Collaborative Inquiry - Emancipatory Research - Iterative Research - User-led Design | *Community*   - Community - Lived experience - Consumer - Public - Patient - Participant - Parent - Family - Stakeholder - Caregiver - End-User - Client - Advocate   *Involvement*   - Collaboration - Partnership - Consultation - Participation - Involvement - Engagement |
| **PSYCINFO SEARCH TERMS** | Autism Spectrum Disorders/  (autis* or asperger* or pervasive development*).mp. | Action Research/ or  Inclusive/ or  Patient participation/ or  Consumer participation/  Community-Based Participatory Research.af  (participatory* or collaborative inquiry or action research or co research* or user-led design).af  (research adj5 (inclusiv* or emancipatory or iterati*)).af  exp Ethics/ | ((community* or lived experience or consumer* or public* or patient* or participant* or parent or parents or parental or family or stakeholder* or caregiver* or end user* or client* or advoca*) adj5 (researcher* or investigator*) adj5 (collaborat* or partner* or consult* or participat* or involve* or engage*)).af. |
| **SCOPUS SEARCH TERMS** | (TITLE-ABS-KEY ( autis* OR asperger* OR pervasive AND development* ) ) | (ALL ( community AND based AND participatory AND research ) OR ALL ( participatory* OR collaborative AND inquiry OR action AND research OR co AND research* OR user AND led AND design ) OR ALL ( ( research ) W/5 ( inclusiv* OR emancipatory OR iterati* ) ) | ALL ( ( ( community* OR lived AND experience OR consumer* OR public* OR patient* OR participant* OR parent OR parents OR parental OR family OR stakeholder* OR caregiver* OR end AND user* OR client* OR advoca* ) AND ( researcher* OR investigator* ) AND ( collaborat* OR partner* OR consult* OR participat* OR involve* OR engage* ) ) ) |
| **PUBMED SEARCH TERMS** | (Autism Spectrum Disorder[MeSH Major Topic]) OR (autis* OR asperger* OR pervasive development*) | ((((Patient participation[MeSH Major Topic]) OR (Community Participation[MeSH Major Topic])) OR (Community-Based Participatory Research)) OR ((participatory*[Title/Abstract] OR collaborative inquiry[Title/Abstract] OR action research[Title/Abstract] OR co research*[Title/Abstract] OR "user-led design"))) OR ((((Autism Spectrum Disorder[MeSH Major Topic]) OR (autis* OR asperger* OR pervasive development*)) AND (((((((((Patient participation[MeSH Major Topic]) OR (Community Participation[MeSH Major Topic])) OR (Community-Based Participatory Research)) OR (participatory*[Title/Abstract])) OR (collaborative inquiry[Title/Abstract])) OR (action research[Title/Abstract])) OR (co research*[Title/Abstract])) OR ("user-led design")) OR (research AND (inclusiv* OR emancipatory OR iterati*)))))) | (((community*[Title/Abstract] OR lived experience[Title/Abstract] OR consumer*[Title/Abstract] OR public*[Title/Abstract] OR patient*[Title/Abstract] OR participant*[Title/Abstract] OR parent[Title/Abstract] OR parents[Title/Abstract] OR parental[Title/Abstract] OR family[Title/Abstract] OR stakeholder*[Title/Abstract] OR caregiver*[Title/Abstract] OR end user*[Title/Abstract] OR client*[Title/Abstract] OR advoca*) AND (researcher*[Title/Abstract] OR investigator*) AND (collaborat*[Title/Abstract] OR partner*[Title/Abstract] OR consult*[Title/Abstract] OR participat*[Title/Abstract] OR involve*[Title/Abstract] OR engage*))) |
| **MEDLINE SEARCH TERMS** | Autism Spectrum Disorders/  (autis* or asperger* or pervasive development*).mp. | Action Research/ or  Inclusive/ or  Patient participation/ or  Consumer participation/  Community-Based Participatory Research.af  (participatory* or collaborative inquiry or action research or co research* or user-led design).af  (research adj5 (inclusiv* or emancipatory or iterati*)).af  exp Ethics/ | ((community* or lived experience or consumer* or public* or patient* or participant* or parent or parents or parental or family or stakeholder* or caregiver* or end user* or client* or advoca*) adj5 (researcher* or investigator*) adj5 (collaborat* or partner* or consult* or participat* or involve* or engage*)).af. |
| **EMBASE SEARCH TERMS** | autism/  (autis* or asperger* or pervasive development*).mp. | Action Research/ or  Patient participation/ or  Ethics/  Community-Based Participatory Research.af  (participatory* or collaborative inquiry or action research or co research* or user-led design).af  (research adj5 (inclusiv* or emancipatory or iterati*)).af | ((community* or lived experience or consumer* or public* or patient* or participant* or parent or parents or parental or family or stakeholder* or caregiver* or end user* or client* or advoca*) adj5 (researcher* or investigator*) adj5 (collaborat* or partner* or consult* or participat* or involve* or engage*)).af. |
| **ERIC SEARCH TERMS** | Autism  autis* or asperger* or pervasive development* | Action Research/ or  Ethics/  Community-Based Participatory Research.af  (participatory* or collaborative inquiry or action research or co research* or user-led design).af  (research N/5 (inclusiv* or emancipatory or iterati*)).af | ((community* or lived experience or consumer* or public* or patient* or participant* or parent or parents or parental or family or stakeholder* or caregiver* or end user* or client* or advoca*) AND (researcher* or investigator*) AND (collaborat* or partner* or consult* or participat* or involve* or engage*)).af. |

Table S2

Coding scheme for obtaining general article information.

| **Code** | **Description** | **Rationale** |
| --- | --- | --- |
| Journal name | Open text field | To examine whether participatory autism research was more likely to be published in certain journals. |
| Type of empirical work | - Qualitative - Quantitative - Mixed methods | To examine whether certain types of research designs were more likely to engage in community involvement than other types. |
| Language Use | - Identity-First - Person-First - Mixed of both - Others | To examine whether the level of participation influences language use. |
| Country | Country where the study was conducted | To examine where studies that involved community members took place. |
| Research area/discipline | Classification came from the IACC Strategic Plan, adapted and employed in den Houting & Pellicano (2019).   - Screening and diagnosis - Biology - Genetic and environmental factors - Interventions - Services and supports - Lifespan - Infrastructure and prevalence | To examine whether certain research areas were more likely to engage in participatory approaches than other areas. |
| Funding information | Classification adapted from Price et al. (2018)   - Industry - Trust - Governmental - University - Service providers - None | To examine whether studies supported by certain types of funders were more likely to engage in community involvement than other types. |

Table S3

Coding scheme for obtaining detailed information on participatory approaches.

| **Code** | **Description** | **Rationale** |
| --- | --- | --- |
| Types of community members involved | - Autistic adults - Autistic researchers - Autistic young people - Parents of Autistic adults - Parents of Autistic young people - Parents of Autistic people (unspecified age) - Family members (unspecified connection) - Clinicians/Healthcare providers - Educators - Professionals (unspecified specialty) - Policymakers - Funders - Patient/Advocacy/Community organisations - Others - Insufficient information | To examine the frequency in which certain groups of community members were involved in the research. |
| Breadth of community involvement | What did community members do?  Classification was adapted from Khodyakov et al. (2013) and den Houting et al. (2021).   - Developing community-based theories of the research - Grant proposal writing - Background research - Choosing research method - Designing study - Developing sampling procedures - Designing/Modifying interview schedules and/or survey questions - Providing inputs on public-facing materials - Developing/Adapting intervention - Recruiting study participants - Implementing the intervention - Collecting primary data - Analysing data - Interpreting study findings - Writing reports/journal articles - Giving presentations at meetings/conferences - Insufficient information | To examine the frequency in which certain aspects of a research project involved community members. |
| Level of participation | Classification was based on Arnstein (1969)   - Community-controlled - Community-led - Co-producing - Engaging - Consulting - Informing - Educating - Coercing - Insufficient information | To examine the frequency in which community involvement was conducted at each level of participation. |

Table S4

This table shows how detailed coding scheme was transformed to a broad coding scheme for the groups of community members.

| **Detailed coding scheme** | **Broad coding scheme** |
| --- | --- |
| Autistic adults | Autistic people |
| Autistic young people |  |
| Autistic researchers |  |
| Parents of Autistic adults | Families of autistic people |
| Parents of Autistic young people |  |
| Parents of Autistic people (unspecified age) |  |
| Family members (unspecified connection) |  |
| Clinicians/Healthcare providers | Professionals |
| Educators |  |
| Professionals (unspecified specialty) |  |
| Policymakers | Administration |
| Funders |  |
| Patients/Advocacy/Community organisation |  |
| Insufficient information | Insufficient information |

Table S5

This table shows how detailed coding scheme was transformed to a broad coding scheme for the breadth of involvement.

| **Detailed coding scheme** | **Broad coding scheme** |
| --- | --- |
| Developing community-based theories | Conceptualisation |
| Grant proposal writing |  |
| Background research |  |
| Choosing research method | Design |
| Designing study |  |
| Developing sampling procedures |  |
| Designing/Modifying interview schedules and/or survey questions |  |
| Providing inputs on public-facing materials |  |
| Developing/Adapting intervention |  |
| Recruiting study participants | Implementation |
| Implementing intervention |  |
| Collecting primary data |  |
| Analysing data | Analysis |
| Interpreting study findings |  |
| Writing reports/journal articles | Dissemination |
| Giving presentations at meetings/conferences |  |
| Insufficient information | Insufficient information |

Table S6

Authors’ review of their participatory approach based on Staniszewska et al.’s (2017) Guidance for Reporting Involvement of Patients and the Public Version 2 (Short Form)

| **1. Aim**  Report the aim of the study | The aim of this review was to examine the extent and nature of participatory approaches within autism research. The involvement of Autistic researcher (JdH) and Autistic community members (TH, HW-S, and MR) was deemed necessary for two main reasons:   1) This is a project about community involvement hence should include community involvement. 2) Autistic people should have a say in deciding whether or not certain participatory approach is inclusive. |
| --- | --- |
| **2. Methods**  Provide a clear description of the methods used for community involvement in the study | DT and EP conceived the initial idea for the review and subsequently brought on JdH, who contributed deep academic expertise in participatory autism research alongside advocacy experience as an Autistic activist promoting such approaches, and TH, who brought extensive experience in community engagement and partnerships. Together, DT, EP, JdH, and TH developed the review protocol, including developing the specific research questions and the search terms. MR and HW-S were then brought on to contribute to abstract and full-text screening as part of their Autism CRC research internship programme, through which they gained insight into the research process. After their internship concluded, MR and HW-S were remunerated for their time to complete screening. DT, MR and HW-S met regularly to discuss and resolve discrepancies in screening decisions. JdH, TH, MR, and HW-S all contributed to data interpretation and manuscript review. The team jointly submitted an abstract to present this work at an international conference. |
| **3. Results**  Outcomes – Report the results of community involvement in the study, including both positive and negative outcomes | JdH, TH, HW-S, and MR contributed to the study in several ways, including:   - Designing review protocol - Screening titles and abstracts - Screening full texts - Interpreting results - Initiating important discussions around greater community involvement in research topics that are more closely aligned with community priorities - Providing feedback on drafts of manuscript |
| **4. Discussion**  Outcomes – Comment on the extent to which PPI influenced the study overall. Describe both positive and negative effects. | The involvement of JdH and TH shaped this review from the protocol stage onward, which we believe strengthened both the framing of our research questions and our interpretation of findings.  Two outcomes were particularly notable. First, our team combined academic and experiential expertise in a way that informed how we conceptualised "participation" itself: JdH's dual positioning as both a participatory autism researcher and an Autistic activist meant that decisions about scope and interpretation were continually checked against both empirical and lived-experience standards, while TH's grounding in community partnerships brought a practical, on-the-ground perspective to how we read the included studies' claims of community involvement. Second, involving MR and HW-S through the Autism CRC research internship programme gave two community co-researchers structured exposure to systematic review methodology.  Overall, the team did not identify any substantial negative effects of this collaborative approach on the conduct or outcomes of the review. |
| **5. Reflections**  Critical perspective – Comment critically on the study, reflecting on the things that went well and those that did not, so others can learn from this experience. | This project gave us the opportunity to apply our own review's findings to a critical reflection on our collaborative process. Several factors contributed to a positive working relationship within the team. MR and HW-S developed a strong rapport with the wider team over the course of their internship and were understanding when aspects of the project took longer than anticipated. Their commitment extended beyond the agreed internship hours, and thanks to available funding support, EP and DT were able to compensate them for this additional contribution. Similarly, TH and JdH's existing close working relationship facilitated smooth collaboration.  At the same time, our experience illustrated some of the practical challenges that participatory approaches can encounter within the realities of academic careers. Partway through the project, both DT and EP transitioned into new roles – DT to another fellowship and EP to another institution – which affected the team's capacity to sustain the depth of involvement originally intended, particularly during coding. As a result, coding was undertaken predominantly by DT, drawing on her prior experience with community-engaged projects, including a similar review reported in Tan et al. (2025). While this provided some methodological continuity, it represented a departure from the broader, more distributed involvement we had intended.  Reflecting on this, if we were to apply our own review's framework to rate our level of participation, we would characterise it as "engaging" rather than a deeper form of collaboration or co-production – a candid acknowledgment that staffing changes and the structure of academic careers can constrain participatory goals even when the team's underlying commitment to inclusive practice remains strong. |

Table S7

Community involvement across different levels of participation and different groups of community members.

|  | **Levels of participation, no. of articles (%)**^†^ | | | | | | |
| --- | --- | --- | --- | --- | --- | --- | --- |
| **Community members** | **Informing** | **Consulting** | **Engaging** | **Co-producing** | **Community Led** | **Community Controlled** | **Insufficient information** |
| Autistic people | 31 (8%) | 77 (20%) | 49 (13%) | 63 (16%) | 17 (4%) | 5 (1%) | 14 (4%) |
| Families | 26 (7%) | 51 (13%) | 31 (8%) | 24 (6%) | 4 (1%) | 0 | 9 (2%) |
| Professionals | 29 (8%) | 56 (15%) | 33 (9%) | 27 (7%) | 2 (0.5%) | 1 (0.3%) | 10 (3%) |
| Administration | 3 (0.8%) | 8 (2%) | 9 (2%) | 4 (1%) | 0 | 0 | 2 (0.5%) |

Table S8

Participatory autism research studies across different countries

|  |  | **Levels of participation, no. of articles (%)**^†^ | | | | | | |
| --- | --- | --- | --- | --- | --- | --- | --- | --- |
| **Country** | **N (%)^*^** | **Informing** | **Consulting** | **Engaging** | **Coproducing** | **Community Led** | **Community Controlled** | **Insufficient information** |
| Argentina | 1 (0.3%) | 0 | 1 (100%) | 0 | 0 | 0 | 0 | 0 |
| Australia | 52 (14%) | 6 (12%) | 13 (25%) | 10 (19%) | 16 (31%) | 3 (6%) | 1 (2%) | 3 (6%) |
| Bangladesh | 1 (0.3%) | 0 | 0 | 1 (100%) | 0 | 0 | 0 | 0 |
| Belgium | 2 (0.5%) | 0 | 2 (100%) | 0 | 0 | 0 | 0 | 0 |
| Brazil | 3 (0.8%) | 0 | 2 (67%) | 0 | 0 | 0 | 0 | 1 (33%) |
| Canada | 31 (8%) | 6 (20%) | 8 (26%) | 11 (35%) | 5 (16%) | 0 | 0 | 1 (3%) |
| Costa Rica | 1 (0.3%) | 0 | 1 (100%) | 0 | 0 | 0 | 0 | 0 |
| Finland | 3 (0.8%) | 0 | 2 (67%) | 1 (33%) | 0 | 0 | 0 | 0 |
| France | 4 (1%) | 1 (25%) | 2 (50%) | 0 | 0 | 0 | 0 | 1 (25%) |
| Germany | 3 (0.8%) | 0 | 0 | 2 (67%) | 1 (33%) | 0 | 0 | 0 |
| Hong Kong | 2 (0.5%) | 0 | 1 (50%) | 0 | 1 (50%) | 0 | 0 | 0 |
| Ireland | 3 (0.8%) | 1 (33%) | 0 | 0 | 0 | 1 (33%) | 0 | 1 (33%) |
| Israel | 4 (1%) | 1 (25%) | 1 (25%) | 0 | 0 | 1 (25%) | 0 | 1 (25%) |
| Italy | 2 (0.5%) | 2 (100%) | 0 | 0 | 0 | 0 | 0 | 0 |
| Jordan | 1 (0.3%) | 0 | 1 (100%) | 0 | 0 | 0 | 0 | 0 |
| Mexico | 1 (0.3%) | 1 (100%) | 0 | 0 | 0 | 0 | 0 | 0 |
| Netherlands | 4 (1%) | 1 (25%) | 2 (50%) | 0 | 0 | 1 (25%) | 0 | 0 |
| New Zealand | 2 (0.5%) | 0 | 0 | 0 | 1 (50%) | 0 | 1 (50%) | 0 |
| Poland | 1 (0.3%) | 0 | 1 (100%) | 0 | 0 | 0 | 0 | 0 |
| South Africa | 1 (0.3%) | 1 (100%) | 0 | 0 | 0 | 0 | 0 | 0 |
| South Korea | 1 (0.3%) | 0 | 1 (100%) | 0 | 0 | 0 | 0 | 0 |
| Spain | 3 (0.8%) | 1 (33%) | 1 (33%) | 1 (34%) | 0 | 0 | 0 | 0 |
| Sweden | 4 (1%) | 1 (25%) | 2 (50%) | 0 | 0 | 0 | 0 | 1 (25%) |
| Taiwan | 3 (0.8%) | 1 (33%) | 1 (33%) | 1 (34%) | 0 | 0 | 0 | 0 |
| Turkey | 1 (0.3%) | 1 (100%) | 0 | 0 | 0 | 0 | 0 | 0 |
| UK | 122 (32%) | 17 (14%) | 33 (27%) | 28 (23%) | 24 (20%) | 7 (6%) | 2 (2%) | 11 (9%) |
| UAE | 1 (0.3%) | 1 (100%) | 0 | 0 | 0 | 0 | 0 | 0 |
| USA | 124 (32%) | 13 (10%) | 45 (36%) | 23 (19%) | 26 (21%) | 5 (4%) | 1 (1%) | 11 (9%) |
| Vietnam | 1 (0.3%) | 1 (100%) | 0 | 0 | 0 | 0 | 0 | 0 |

***Note.*** *Percentage out of the 382 included articles.

^†^Percentage out of the country’s total number of articles.

Table S9

Types of research methods employed in studies grouped by levels of participation.

|  | **Levels of participation, no. of articles (%)**^†^ | | | | | | |
| --- | --- | --- | --- | --- | --- | --- | --- |
| **Research methods** | **Informing** | **Consulting** | **Engaging** | **Coproducing** | **Community Led** | **Community Controlled** | **Insufficient information** |
| Qualitative | 43 (11%) | 55 (14%) | 37 (9.7%) | 37 (9.7%) | 12 (3.1%) | 5 (1.3%) | 0 |
| Quantitative descriptive | 3 (0.8%) | 33 (8.6%) | 11 (2.9%) | 17 (4.5%) | 3 (0.8%) | 0 | 10 (2.6%) |
| Quantitative randomised controlled trial | 1 (0.3%) | 5 (1.3%) | 3 (0.8%) | 1 (0.3%) | 0 | 0 | 2 (0.5%) |
| Quantitative non-randomised trail | 1 (0.3%) | 4 (1.0%) | 2 (0.5%) | 1 (0.3%) | 0 | 0 | 1 (0.3%) |
| Mixed methods | 4 (1.0%) | 8 (2.1%) | 8 (2.1%) | 2 (0.5%) | 1 (0.3%) |  | 2 (0.5%) |
| Multi methods* | 4 (1.0%) | 15 (3.9%) | 17 (4.5%) | 16 (4.2%) | 2 (0.5%) | 0 | 4 (1.0%) |

***Note.*** *Studies that collected qualitative and quantitative data which were not analysed using mixed methods.

^†^Percentage out of the total number of articles using each research method.

Table S10

Language use in studies involving community members grouped by levels of participation.

|  |  | **Levels of participation, *n* (%)**^†^ | | | | | | |
| --- | --- | --- | --- | --- | --- | --- | --- | --- |
| **Language** | ***N* (%)^*^** | **Informing**  **(*n* = 56)** | **Consulting**  **(*n* = 120)** | **Engaging**  **(*n* = 78)** | **Coproducing**  **(*n* = 74)** | **Community Led**  **(*n* = 18)** | **Community Controlled**  **(*n* = 5)** | **Insufficient information**  **(*n* = 31)** |
| Mostly identity-first | 207 (54%) | 8 (14%) | 51 (43%) | 47 (60%) | 59 (80%) | 16 (89%) | 5 (100%) | 21 (68%) |
| Mostly person-first | 162 (42%) | 47 (84%) | 60 (50%) | 31 (40%) | 14 (19%) | 1 (5.5%) | 0 | 9 (29%) |
| Mix of both | 13 (4%) | 1 (2%) | 9 (7%) | 0 | 1 (1%) | 1 (5.5%) | 0 | 1 (3%) |

***Note.*** ^*^Percentage out of the 382 included articles.

^†^Percentage out of the total number of articles under each level of participation.
